# AI-Assisted LVEF Assessment Using a Handheld Ultrasound Device: A Single-Center Comparative Study Against Cardiac Magnetic Resonance Imaging

**DOI:** 10.3390/jcm14134708

**Published:** 2025-07-03

**Authors:** Giovanni Bisignani, Lorenzo Volpe, Andrea Madeo, Riccardo Vico, Davide Bencardino, Silvana De Bonis

**Affiliations:** 1 Cardiology Division, “G. Ferrari” Civil Hospital of Castrovillari, 87012 Cosenza, Italy; andreamadeo83@gmail.com (A.M.); riccardovico@libero.it (R.V.); 2 Radiodiagnostics Division, “G. Ferrari” Civil Hospital of Castrovillari, 87012 Cosenza, Italy; bencardino.davide@gmail.com; 3 Cardiology Division, Civil Hospital of Corigliano-Rossano, 87064 Cosenza, Italy; silvanadebonis1968@gmail.com

**Keywords:** echocardiography, cardiac magnetic resonance, cardiovascular imaging

## Abstract

**Background/Objectives:** Two-dimensional echocardiography (2D echo) is widely used for assessing left ventricular ejection fraction (LVEF). This single-center comparative study aims to evaluate the accuracy of LVEF measurements obtained using the AI-assisted handheld ultrasound device Kosmos against cardiac magnetic resonance (CMR), the current gold standard. **Methods:** A total of 49 adult patients undergoing clinically indicated CMR were prospectively enrolled. AI-based LVEF measurements were compared with CMR using the Wilcoxon signed-rank test, Pearson correlation, multivariable linear regression, and Bland–Altman analysis. All analyses were performed using STATA v18.0. **Results:** Median LVEF was 57% (CMR) vs. 55% (AI-Echo), with no significant difference (*p* = 0.51). Strong correlation (r = 0.99) and minimal bias (1.1%) were observed. **Conclusions:** The Kosmos AI-based autoEF algorithm demonstrated excellent agreement with CMR-derived LVEF values. Its speed and automation make it promising for bedside assessment in emergency departments, intensive care units, and outpatient clinics. This study aims to fill the gap in current clinical evidence by evaluating, for the first time, the agreement between LVEF measurements obtained via Kosmos’ AI-assisted autoEF and those from cardiac MRI (CMR), the gold standard for ventricular function assessment. This comparison is critical for validating the reliability of portable AI-driven echocardiographic tools in real-world clinical practice. However, these findings derive from a selected population at a single Italian center and should be validated in larger, diverse cohorts before assuming global generalizability.

## 1. Introduction

Two-dimensional echocardiography (2D echo) is one of the most widely utilized imaging techniques [1] due to its accessibility, ease of use, affordability, portability for bedside application, and repeatability in both elective and emergency settings [1,2]. These features make it a valuable tool for the diagnosis and prognosis of various cardiac diseases [3]. Quantitative methods are preferred over qualitative visual estimations for calculating left ventricular ejection fraction (LVEF), as they reduce variability and enhance precision and accuracy [4]. The biplane disc summation method (modified Simpson’s rule) for calculating left ventricular volumes is the current standard recommended by the American Society of Echocardiography and the European Association of Cardiovascular Imaging [5,6]. In recent years, portable echocardiographic devices have gained traction in clinical practice [7]. Often referred to as the “stethoscope of the future,” these devices facilitate use by non-expert cardiologists in diverse clinical contexts [8]. More recently, machine learning techniques—commonly referred to as artificial intelligence (AI)—have been employed to automatically delineate endocardial borders on routine 2D echocardiographic images, enabling rapid LVEF estimation [9].

The autoEF approach, developed by the U.S. medical equipment company Kosmos (EchoNous), leverages AI-driven programming and recognition models trained on extensive databases containing thousands of echocardiographic images with varying qualities and pathologies, annotated by expert investigators [10]. Kosmos is the first patented clinical tool to integrate hybrid point-of-care ultrasound (POCUS) capabilities, designed to enhance bedside diagnostic confidence and facilitate the rapid calculation of LVEF [11]. In March 2020, the Kosmos platform became the first AI-assisted handheld ultrasound device to receive U.S. Food and Drug Administration (FDA) approval for clinical use, as well as European CE mark approval [12]. Portable LVEF measurements are designed to be used at the bedside, reducing waiting times and improving diagnostic efficiency. These tools offer significant advantages in terms of accessibility, speed, and ease of use, even in clinical settings with limited resources.

Several studies have since validated the reliability and diagnostic accuracy of this innovative AI-assisted device for LVEF calculation when compared with traditional echocardiography [13]. These studies have shown that portable AI devices provide LVEF measurements comparable to those obtained with traditional echocardiography. For example, a multicenter study in Japan reported an intraclass correlation (ICC) of 0.81 between LVEF measured with a portable AI device and the standard biplane method, with no systematic, clinically significant differences. In another study, the autoEF algorithm of a portable device showed an ICC of 0.85 compared to the manual Simpson biplane method, with a sensitivity of 88% and a specificity of 87% in detecting reduced LVEF (≤50%).

The accuracy of measurements may vary based on operator experience. However, studies have shown that even less experienced users can obtain reliable results with portable AI devices. In a study of 449 patients, the diagnostic accuracy of AI was high for both novices (92.8% sensitivity) and experts (92.3% specificity) [14]. Portable LVEF devices are particularly useful in clinical settings where access to traditional ultrasound is limited, such as emergency rooms, intensive care units, or home care. Their ability to provide rapid and reliable LVEF measurements allows for the timely assessment of cardiac function, which is critical for the management of patients with heart failure or other cardiovascular diseases.

Despite advances, it is important to consider that the accuracy of portable devices can be affected by factors such as image quality, acquisition technique, and individual patient characteristics. Therefore, it is essential that healthcare professionals are adequately trained in the use of these devices and in the interpretation of results.

Magnetic resonance imaging (CMR), with its superior contrast resolution, sufficient spatial resolution, and ability to perform three-dimensional evaluations, is widely regarded as the gold standard for assessing ventricular volumes [15]. CMR overcomes the geometric assumptions inherent in ultrasound methods [16]. However, direct comparisons between portable echocardiographic AI devices and CMR are limited.

A study evaluated the accuracy of a fully automated AI algorithm (autoEF) in calculating LVEF, comparing it with the modified Simpson method in echocardiography and with CMR as a reference. The results showed a strong correlation between autoEF and CMR (R = 0.89), with low interobserver variability (intraclass correlation coefficient = 1.00). In contrast, the modified Simpson method showed higher variability and lower correlation with CMR. Furthermore, autoEF underestimated LVEF (bias: 2.2%) while the modified Simpson method overestimated it (bias: −2.2%) [17]. Another study compared the autoEF with manual EF measurement in 242 patients, using CMR as a reference. The correlation between AI and CMR was r = 0.89, similar to that between manual EF measurement and CMR (r = 0.89). Bland-Altman analysis showed a bias of 3.63 ± 7.40% for AI and 0.33 ± 7.52% for manual EF measurement, indicating that both methods are comparable in accuracy [18].

An additional study compared the repeatability of LVEF measurements obtained by AI and manually in CMR. The automatic measurements showed a high interobserver correlation (ICC = 0.80 for LVEF), with minimal differences between repeated measurements (*t* test *p* = 0.73) [19]. Similar to the above studies, the aim of this study was to evaluate the concordance between LVEF measurements obtained using the Kosmos AI-assisted autoEF algorithm and those derived from CMR, thereby assessing the reliability and accuracy of the AI-based method.

Therefore, the objective of this study is to assess the concordance between LVEF values obtained using the Kosmos AI-assisted autoEF algorithm and those derived from cardiac MRI. While prior studies have compared autoEF against standard echocardiographic techniques, few have rigorously validated it against CMR, and even fewer have done so using portable, point-of-care devices. This study directly addresses this gap, aiming to support the broader clinical adoption of handheld AI-powered echocardiography for reliable, bedside cardiac function assessment.

## 2. Materials and Methods

The study enrolled 49 consecutive adult patients referred for cardiac MRI. All participants underwent an additional echocardiographic evaluation using the Kosmos device, performed by a single experienced cardiologist. A formal sample size calculation was not performed, as this was an exploratory, single-center validation study. The sample size was determined based on the number of consecutive eligible patients undergoing scheduled cardiac MRI during the study period. For 35 patients (71.4%), CMR and echocardiographic assessments were conducted on the same day. In the remaining 14 patients (28.6%), the exams were performed within 72 h of each other, primarily due to logistical scheduling constraints. No clinical events or therapeutic interventions occurred between the two assessments that could influence LVEF measurements. Patients were included if over 18 and hemodynamically stable. Exclusion criteria included atrial fibrillation, frequent ectopy, severe comorbidities, pregnancy, breastfeeding, and patients under 18. All participants provided written informed consent. The study complied with local ethics guidelines and the Declaration of Helsinki.

**Figure 1:**
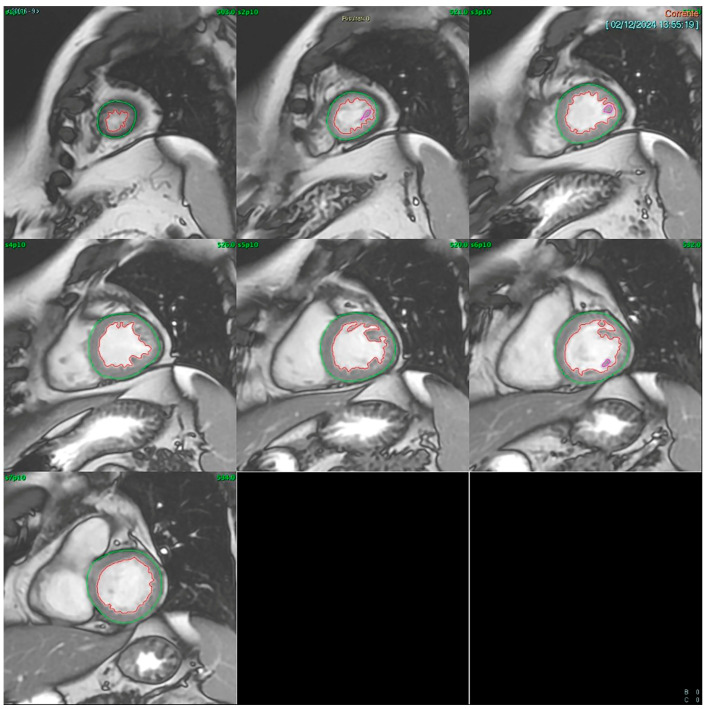
Automatic contouring of the left ventricle on an SSFP contiguous diastolic short-axis cine images stack from base to apex.

**Cardiac MRI (CMR):** CMR was conducted using a GE SIGNA™ 1.5T system (General Electric (Healthcare), Boston, MA, USA). Multishot fast spin-echo sequences were acquired along the short and long axes for morphological evaluation, while cine-MRI fast gradient echo sequences in respiratory apnea were used for dynamic assessment. Short-axis images included 8–9 contiguous slices with 10 mm thickness, covering the entire left ventricle and capturing at least 20 cardiac phases per slice. LVEF was calculated using the Simpson rule (method of disks) applied to a contiguous short-axis cine stack acquired via SSFP sequences. The left ventricular end-diastolic and end-systolic volumes were manually traced in each slice from base to apex (Figure 1). All analyses were performed offline by a single experienced CMR reader using blinded echocardiography results.

**Echocardiography:** Echocardiography was conducted using a novel handheld ultrasound device (Kosmos, EchoNous, Inc., Redmond, WA, USA) equipped with a 2- to 5-MHz phased-array transducer also suitable for pulmonary and abdominal ultrasound applications. The Kosmos system represents an advanced and versatile solution for portable ultrasound, combining high-quality hardware with AI software to improve efficiency and accuracy in clinical assessments. The high-resolution 1920 × 1200 pixel display provides clear and detailed images for accurate evaluation of ultrasound images. The device is equipped with 128 GB of internal memory, sufficient to store numerous exams and images.

AI features include Auto Preset (automatic optimization of presets during scans), Auto Doppler (automatic positioning of the Doppler gate for PW and TDI), AI FAST (automatic identification of anatomical windows), and Auto EF (automatic calculation of ejection fraction and stroke volume). Kosmos is compatible with iOS (iPad Air and Pro) and Android (Samsung Galaxy Tab Active Pro, Lenovo Tab P11 Pro, Xiaomi Pad 6) devices, offering flexibility in device choice. It supports image export via USB-C in JPEG, MP4, and DICOM formats, facilitating integration with healthcare information systems. It also enables data backup to remote devices, ensuring the safety and protection of sensitive information.

**Figure 2:**
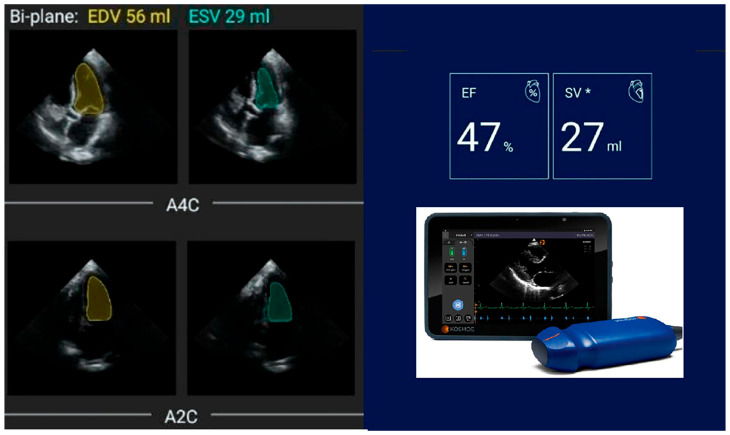
Following the recommendations of the American Society of Echocardiography and the European Association of Cardiovascular Imaging (modified Simpson’s rule), AI algorithm first identifies end-systolic (ED) and end-diastolic (ES) frames in the A4C and A2C clips, then segments LV in all four frames (A4C ED and ES frames, A2C ED and ES frames) to automatically calculate LVEF in just 5 s. * Stroke Volume.

In this study, LVEF was calculated by AI-assisted autoEF. All participants were scanned by the same cardiologist to ensure consistency. The Kosmos device uniquely integrates both pulsed wave and continuous wave capabilities and supports cardiac, abdominal, and pulmonary imaging, making it versatile for diverse clinical scenarios. The autoEF algorithm leverages the concept of pattern recognition from AI theory, designed to mimic human reasoning and learn from past experiences. Using deep learning algorithms (software version 3.0.1.78), the device facilitates image acquisition and automatically calculates LVEF. It captures apical four-chamber (A4C) and apical two-chamber (A2C) views of the heart, each recorded over five seconds, with the patient positioned in the left lateral decubitus posture. The algorithm works without the need for a gated electrocardiogram. In alignment with the recommendations of the American Society of Echocardiography and the European Association of Cardiovascular Imaging (modified Simpson’s rule), the AI algorithm identifies end-systolic (ES) and end-diastolic (ED) frames in the A4C and A2C clips. It then segments the left ventricle in all four frames (A4C ED and ES, A2C ED and ES) to calculate LVEF automatically within five seconds (Figure 2). The results were saved immediately without manual corrections or tracing. The algorithm does not assess other functional parameters such as global longitudinal strain (GLS); thus, the study was limited to EF assessment.

**Statistical Methods:** Descriptive statistics were presented as counts (percentages) for categorical variables and as medians (interquartile range, IQR) for continuous variables. The Shapiro–Wilk test was employed to assess the normality of the distribution of left ventricular ejection fraction values measured by cardiac magnetic resonance (LVEF-CMR) and artificial intelligence-based echocardiography (LVEF AI-Echo). Comparisons between LVEF-CMR and LVEF AI-Echo measurements were performed using the non-parametric Wilcoxon signed-rank test. Univariable and multivariable linear regression analyses were conducted to evaluate the correlation between LVEF-CMR and LVEF AI-Echo measurements, with LVEF-CMR serving as the dependent variable and LVEF AI-Echo as the independent predictor, excluding the intercept. The multivariable analysis accounted for age, sex, and same-day investigation as covariates. Agreement between the two LVEF measurement methods was assessed using the Bland–Altman analysis. A *p*-value of <0.05 was considered statistically significant for all analyses, which were conducted using STATA software version 18.0 (StataCorp LP, College Station, TX, USA).

## 3. Results

**Patient Characteristics:** A total of 49 patients were analyzed, with 32.7% being women and a median age of 56.9 years (IQR, 41.5–64.7). Patients were referred for cardiac magnetic resonance imaging for myocardial infarction with non-obstructive coronary arteries (MINOCA) (46.7%), myocarditis and pericarditis (32.2%), hypertrophic cardiomyopathy (18.7%), and cardiac mass or tumor (2.4%). For the majority of participants (71.4%), LVEF measurements using CMR and AI-Echo were conducted on the same day (Table 1). The median LVEF values were 57% (IQR, 52–64%) for CMR and 55% (IQR, 52–60%) for AI-Echo, with no statistically significant difference observed between the two methods (*p* = 0.51).

**Figure 3:**
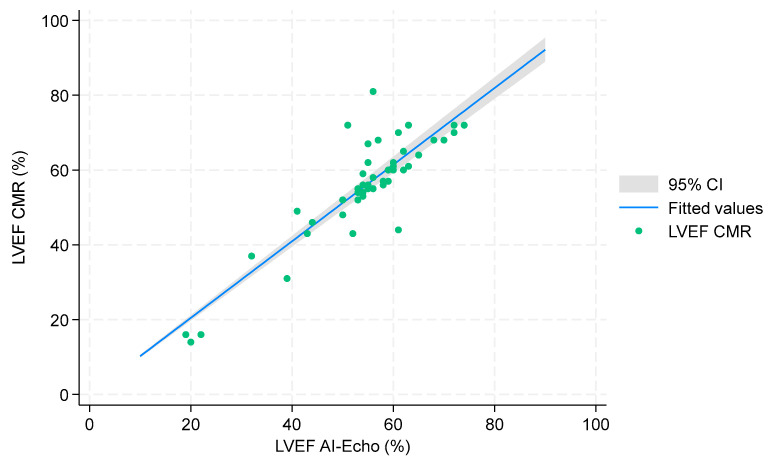
Linear regression between AI-derived and CMR-derived LVEF. Regression equation: LVEF_CMR = 1.042 × LVEF_AI-Echo (95% CI: 0.940–1.144, *p* < 0.001). R^2^ = 0.986.

**Figure 4:**
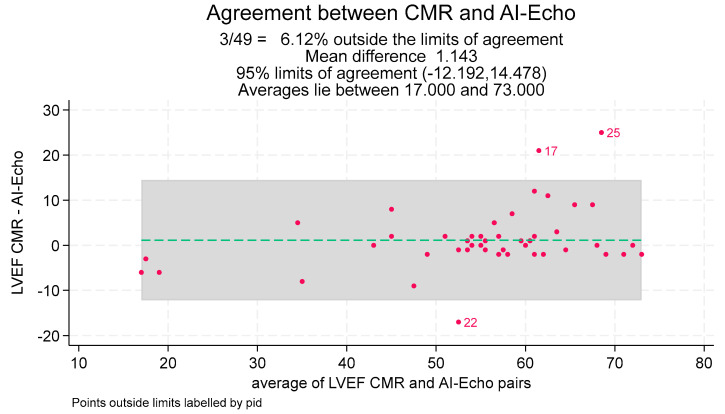
Results of Bland–Altman analysis with points outside the 95% limits of agreement (grey area) between CMR and AI-Echo methods labeled by patient ID 17, 22, and 25. LVEF = left ventricular ejection fraction; CMR = cardiac magnetic resonance; AI-Echo= artificial intelligence-based echocardiography.

**Figure 5:**
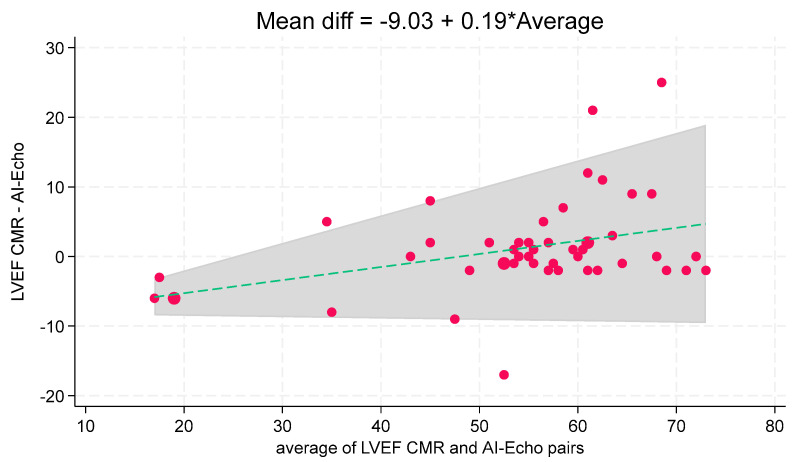
Trend of mean difference between LVEF CMR and LVEF AI-Echo with respect to the average of LVEF CMR and LVEF AI-Echo pairs. LVEF = left ventricular ejection fraction; CMR = cardiac magnetic resonance; AI-Echo= artificial intelligence-based echocardiography.

Univariable linear regression analysis showed a strong correlation between CMR and AI-Echo measurements, with a Pearson’s correlation coefficient of 0.99 and an unadjusted regression coefficient of 1.02 (95% CI, 0.99–1.06). These results were further corroborated by multivariable analysis, yielding an adjusted regression coefficient of 1.04 (95% CI, 0.94–1.14) (Table 2, Figure 3). The Bland–Altman analysis demonstrated good agreement between the two methods, with a mean bias of 1.1% and an agreement range of −12% to +14%. Only three measurements (6.1%) fell outside the limits of agreement (Figure 4). Notably, discrepancies between CMR and AI-Echo measurements were more pronounced at higher LVEF values. Figure 5 illustrates a linear trend showing increasing differences between LVEF-CMR and LVEF AI-Echo as LVEF values rise.

## 4. Discussion

Our study demonstrated, for the first time, that fully automated, AI-assisted LVEF measurement by Kosmos is not only feasible but also highly reliable and comparable to measurements obtained using cardiac MRI. The strong agreement between these imaging methods highlights its potential clinical significance, particularly for hospitalized patients requiring rapid assessments, such as in emergency settings where ease of use and portability are critical [20]. The Kosmos device offers operator guidance for acquiring echocardiographic sections and ensuring image quality, making it suitable as an initial screening tool for large-scale population studies, even by novice users. Initially designed as a learning tool for medical students, the device’s image quality is critical during the training phase, as poor image resolution can hinder skill acquisition [21].

AI-assisted handheld echocardiography devices like Kosmos hold substantial potential to transform cardiovascular care. In clinical practice, they may facilitate early triage in emergency settings, enable rapid bedside decision-making, and support routine monitoring of chronic heart failure patients, particularly in outpatient or home care scenarios. Their portability and ease of use make them ideal for deployment in rural or resource-constrained settings where access to advanced imaging modalities is limited. Additionally, their integration into telemedicine workflows may allow remote acquisition of echocardiographic data by nurses or general practitioners, with automated results transmitted to specialists for interpretation, expanding the reach of cardiac diagnostics. These tools also have promising applications in medical education, serving as training platforms for students and non-cardiologists, supported by AI-guided image acquisition and interpretation. Advances in AI may lead to integration with clinical decision support systems, allowing automatic alerts for abnormal findings or dynamic monitoring of therapy responses. Ultimately, these innovations could streamline diagnostic pathways, reduce healthcare costs, and promote equity in access to cardiovascular imaging.

Future iterations of handheld ultrasound (US) devices will likely incorporate advanced machine learning algorithms to automate image analysis and echocardiographic measurements, further reducing inter-operator variability [22]. This device has the potential to significantly influence bedside treatment decisions and expedite therapeutic interventions [23]. For instance, L. Papadopoulou et al. reported that using portable echocardiography reduced the need for standard echocardiography by 59%, alongside substantial reductions in cost and decision-making time [10]. Portable AI-assisted echocardiography, as a preliminary diagnostic tool before standard echocardiography, is cost-effective and poised to revolutionize current diagnostic strategies in clinical practice [24].

Technological advancements have markedly improved image quality, boosting clinician confidence in using these devices as reliable bedside diagnostic tools [25]. Furthermore, the Kosmos device features a versatile single probe capable of vascular, pulmonary, and abdominal imaging, enhancing its utility across various clinical applications [26]. Currently, its primary application remains the automatic calculation of LVEF, a critical parameter in decision-making for heart failure patients when initiating medical, mechanical, or electrical therapies [27]. Although three-dimensional echocardiography (3DE) provides more accurate LV volume measurements than 2DE, its limited adoption in clinical practice is attributed to higher costs and operational complexity. This underscores the need for reproducible and rapid methods for assessing LV function [28].

Accurate LVEF measurement is essential for clinical decision-making, and our results confirm the reliability of the autoEF algorithm. Manual EF assessments were not included in our analysis, as the device lacks a feature for manual endocardial edge tracking. While users can adjust the edges defined by the autoEF algorithm, this semi-automatic approach would increase processing time and is impractical for bedside applications. Previous studies using other portable ultrasound devices (PUDs) have reported lower concordance between autoEF and CMR-derived EF measurements, likely due to the exclusive use of the A4C view in those studies [29]. In contrast, our study demonstrates a high degree of agreement between the two imaging methods, supported by a multivariable regression analysis with an adjusted regression coefficient of 1.04 (95% CI, 0.94–1.14) (Table 2, Figure 3). Bland–Altman analysis showed good agreement (mean bias: 1.1%), with three cases (6.1%) outside the 95% limits of agreement (Figure 4). These outliers occurred at higher LVEF values, consistent with a slight linear trend observed (Figure 5). The linear regression between AI-Echo and CMR-derived LVEF is expressed by the equation: LVEF_CMR = 1.042 × LVEF_AI-Echo, with 95% CI for the slope: [0.940–1.144]; *p* < 0.001. Discrepancies between CMR and AI-Echo measurements were more pronounced at higher LVEF values, possibly due to challenges in delineating the endocardial contour in the presence of papillary muscles and trabeculae. Conversely, in lower LVEF values, the endocardial contour is more clearly defined.

AI reduces the impact of individual experience, which is particularly important for less experienced operators. Automated algorithms provide more consistent results than manual analysis, reducing intra- and inter-observer variability. Algorithms such as autoEF can determine LVEF in near real time, with performance comparable to magnetic resonance imaging (CMR) while avoiding frequent underestimation or overestimation with subjective “visual” estimation. Reducing the time needed for each examination has clear benefits for high-intensity departments (e.g., emergency room, intensive care). Thanks to the combination of portable ultrasound, it enables diagnosis even in environments with limited access to specialists. AI can analyze echocardiographic data acquired remotely and send the results to specialists, facilitating remote diagnosis. AI systems improve over time through feedback and updates, refining diagnostic accuracy. AI enables the automated analysis of thousands of echocardiographic studies, facilitating epidemiological research or the identification of patient subgroups.

AI offers many advantages but also has important limitations that are essential to consider for informed clinical use. Accuracy is strongly affected by the quality of the ultrasound image. In patients with suboptimal acoustic windows (obesity, COPD, post-surgery), the results may be inaccurate. If the endocardial contours are not well visible, the algorithm may track incorrectly and calculate unrealistic volumes. AI provides a number (the LVEF) but does not interpret the clinical context of the patient (e.g., tachycardia, valvular disease, dyssynchrony). It does not recognize conditions that mimic a normal LVEF (e.g., severe mitral regurgitation with low effective flow). The algorithms are trained on selected data, often from high-volume centers or non-heterogeneous populations. This may lead to lower performance in underrepresented groups (frail elderly, pediatric patients, different ethnicities, and complex heart disease). AI does not currently “learn” from single clinical errors: if an algorithm makes a mistake on a patient, it does not change its behavior unless a central update is planned. Less experienced clinicians may blindly trust AI results, reducing the ability to critically evaluate clinical learning. There is a risk of ignoring obvious errors if active human supervision is not maintained.

This study has several limitations. First, the relatively small sample size (n = 49) limits the statistical power to detect subgroup-specific differences and may affect the generalizability of the results to broader populations. As a feasibility and validation study, our primary goal was to evaluate agreement with CMR rather than to make population-level inferences. Second, the study was conducted in a single center, and all examinations were performed by a single experienced cardiologist. While this minimized operator-dependent variability and enhanced internal consistency, it may have introduced observation or confirmation bias and reduced the study’s external validity. Real-world settings often involve diverse operators with varying levels of expertise. Another limitation is the absence of a direct comparison between AI-derived LVEF and conventional manual biplane measurements, which represent the current clinical standard. This comparison would have strengthened the evaluation of AI diagnostic performance and potential advantages in daily practice. Furthermore, we excluded patients with poor acoustic windows or significant image quality limitations, such as those with obesity, chronic obstructive pulmonary disease, or prior thoracic surgeries. While this was done to ensure optimal conditions for comparison with CMR, it inherently limits the applicability of our results to typical real-world settings, where suboptimal echocardiographic windows are common. Several studies have shown that AI algorithms may underperform in these challenging scenarios due to difficulty in endocardial border detection.

Future research should explore the performance of AI-based handheld ultrasound in multicenter settings involving a wider range of operators, including non-experts such as nurses or junior clinicians. In addition, evaluation in specific subgroups, such as patients with valvular heart disease, atrial fibrillation, or suboptimal imaging conditions, will be essential to assess clinical robustness. Further studies should also investigate the tool’s impact on workflow efficiency, diagnostic decision-making, and clinical outcomes.

## 5. Conclusions

The artificial intelligence-assisted LVEF assessment (autoEF) by Kosmos has demonstrated, for the first time, reliability and accuracy comparable to LVEF calculations obtained via cardiac MRI. This study highlights its potential clinical significance, showcasing a system equipped with probes similar to those of trolley-based systems and capable of delivering rapid, dependable assessments directly at the patient’s bedside in both elective and emergency settings. Furthermore, the device’s ease of use and integrated guidance for precise probe positioning and proper exam execution may expand its applicability, making it accessible even to non-expert echocardiographers and opening new possibilities for its broader utilization.

## Figures and Tables

**Table 1:** Baseline characteristics.

	N = 49
Age (years)	56.9 (41.5–64.7)
Sex (female)	16 (32.7%)
LVEF CMR (%)	57 (52–64)
LVEF AI-Echo (%)	55 (52–60)
Same day investigation	35 (71.4%)

Data are expressed as n (%) and median (interquartile range, IQR). LVEF = left ventricular ejection fraction; CMR = cardiac magnetic resonance; AI-Echo = artificial intelligence-based echocardiography.

**Table 2:** Results of univariable and multivariable linear regression analyses.

	Predictor	Coefficient	Std. Err.	95% CI	*p*-Value	R-Squared
Univariable	LVEF AI-Echo	1.024	0.018	0.989–1.059	<0.001	0.9861
Multivariable		1.042	0.051	0.940–1.144	<0.001	0.9862
	Age	−0.03	0.049	−0.129–0.069	0.548	
	Sex	−0.254	2.185	−4.654–4.146	0.908	
	Same-day investigation	0.814	2.283	−3.784–5.412	0.723	

LVEF = left ventricular ejection fraction; CMR = cardiac magnetic resonance; AI-Echo = artificial intelligence-based echocardiography.

## Data Availability

The original contributions presented in this study are included in the article. Further inquiries can be directed to the corresponding author.
